# Therapeutic Potential of Feline Adipose-Derived Stem Cell Exosomes in the Treatment of Feline Idiopathic Cystitis: A Characterization and Functional Analysis of miRNA Content

**DOI:** 10.7150/ntno.99383

**Published:** 2025-01-01

**Authors:** Andrea Rubini, Federica Zanotti, Danilo Licastro, Giulia Calogero, Gisella Bettini, Cristiana Piccoli, Giuseppe Rubini, Luca Lovatti, Barbara Zavan

**Affiliations:** 1 Ultravet Diagnostics, 40017, San Giovanni in Persiceto, Italy; 2 Department of Cell Biology, Albert Einstein College of Medicine, Bronx, NY 10461, USA.; 3 Department of Translational Medicine, University of Ferrara, 44121, Ferrara, Italy.; 4 Department of Medical Sciences, University of Ferrara, 44121, Ferrara, Italy.; 5 AREA Science Park, 34149, Trieste, Italy.; 6 Clinica Veterinaria Estense, Via Pianelle, 31, 44123 Francolino (Ferrara), Italy.; 7 ZetaRL, Castello D'Argile, Bologna, Italy.

**Keywords:** Feline Adipose-derived Mesenchymal Stem Cells (fADSCs), Exosomes, miRNAs, Veterinary Medicine, Innovative therapy

## Abstract

Feline Idiopathic Cystitis (FIC), is a chronic lower urinary tract condition in cats analogous to PBS/IC in women, which presents significant treatment challenges due to its idiopathic nature. Recent advancements in regenerative medicine highlight the potential of Adipose Tissue-Derived Stem Cells (ADSCs), particularly through their secretome, which includes mediators, bioactive molecules, and extracellular vesicles (EVs). Notably, exosomes, a subset of EVs, facilitate cell-to-cell communication and, when derived from ADSCs, exhibit anti-inflammatory properties and contribute to tissue regeneration. In this work, we aim to characterize the content of exosomes derived from feline ADSCs (fADSCs) to elucidate their mechanisms of action on recipient cells and assess their therapeutic potential for FIC. Exosomes were isolated from fADSCs and their microRNA (miRNA) content sequenced using Illumina technology. Our findings demonstrate that fADSC-derived exosomes harbor miRNAs that can induce regenerative processes, such as cell proliferation, immune modulation, angiogenesis, and anti-inflammatory responses. Key miRNAs identified include fca-miR-221, fca-let-7f-5p, fca-miR-337-5p, fca-miR-542-5p, fca-miR-24-3p, fca-miR-205, and fca-miR-23a, which promote proliferative, angiogenic, differentiation, and regenerative mechanisms. Additionally, miRNAs with anti-inflammatory effects, such as fca-miR-193a-5p and fca-miR-127-3p, and those positively regulating the immune system, including fca-let-7a-5p and fca-miR-chrC1_18846-5p, were identified. Of particular interest, fca-miR-219-5p (has-miR-6766-3p) has been reported to suppress liver fibrosis.These results underline the therapeutic potential of fADSC-derived exosomes in treating FIC and suggest innovative strategies for feline veterinary medicine.

## 1. Background

Feline Idiopathic Cystitis (FIC) is a persistent lower urinary tract disorder in cats, comparable to PBS/IC in women, and poses significant treatment difficulties due to its unknown cause. Advances in regenerative medicine emphasize the promise of Adipose Tissue-Derived Stem Cells (ADSCs), particularly through their secretome, which contains mediators, bioactive molecules, and extracellular vescicles (EVs). Among these, exosomes, a type of EV, play a crucial role in cell-to-cell communication. When sourced from ADSCs, exosomes demonstrate anti-inflammatory effects and promote tissue regeneration 1,2. Currently, it is well recognized that Adipose Tissue-Derived Stem Cells (ADSCs) offer multiple therapeutic benefits for various conditions. Like Bone Marrow-Derived Stem Cells (BMSCs) but easier to isolate, ADSCs show significant potential in regenerative medicine. This potential is linked to the ADSCs' secretome, which comprises mediators, bioactive molecules, and EVs. Numerous studies in regenerative medicine have shown that ADSCs secrete factors that can modulate angiogenesis and anti-inflammatory processes, thereby inducing tissue regeneration. Exosomes are extracellular vesicles with a diameter between 30 and 150 nm, which derived from all types of cells and released into all biological fluids, such as blood plasma, serum, urine, breast milk, colostrum, and conditioned cellular medium in research laboratory 3. Exosomes are involved in cell-to-cell communication and mRNA, miRNA, DNA, lipids, and proteins compose their content, which is selectively taken up by near, or dis-tant target cells, influencing their behavior 4. Recent studies have shown that exosomes derived from ADSCs promote anti-inflammation response reducing inflammation response and play a crucial role in tissue regeneration processes 5. Exosomes can be used as diagnostic, prognostic, and, in some cases, therapeutic treatment in several diseases. It is also reported that exosomes were used as drug-delivery systems in several conditions as an example for neurodegenerative diseases and cancer therapy 6.

Many of the biologic effects of exosomes have been attributed to miRNAs 7. MicroRNAs are small non-coding RNAs that act as post-transcriptional regulators of gene expression, ranging in length from 18 to 22 nucleotides when in their mature form. miRNAs resulted in being involved in complex regulatory networks, basically playing a role in every aspect of the biology of cells and organs 8-9. They affect the gene expression of the cells through silencing. In detail, miRNAs can perfectly match whit mRNA leading to its degradation or they can impartially match whit mRNA strand and block protein translation 9.

On these premises it is understandable that miRNAs taking together to all exosome content allow to regulate complex intracellular pathways. So, exosomes have the potential to be used for therapeutic control and treatment of many diseases 11. In human medicine field they are mainly exploited as predictive markers, 12 even if recent studies have shown that small extracellular vesicles, derived from MSCs, exosomes included, can promote the anti-inflammation response, and play a crucial role in reducing the inflammation in general 13. It is easier to think of a therapeutic use of exosomes when translated to a veterinary field. Indeed, there are many works demonstrated potential use of exosomes as therapy for many pathological states, like fibrosis, atrophy and disease characterized by inflammatory profile. In addition, as for the human medicine field, also in veterinary field, exosomes can be used as diagnostic biomarkers in different medicine branches like oncology and nephrology. Recently, we utilized exosomes derived from dog adipose tissue stem cells to treat hepatopathies in canines, introducing an effective and innovative cell-free therapy to the clinical veterinary field. The exosomes were isolated and characterized for size, distribution, surface markers, and miRNA content via microRNA sequencing. A total of 295 dogs with hepatopathies were treated and monitored for six months to track their biochemical marker levels. The results showed that the exosomes from cADSCs had an average diameter of 91 nm and were positive for eight known exosome markers. Administering these exosomes to dogs with liver-related inflammatory conditions led to recovery and normalization of biochemical parameters of kidney function. In conclusion, exosomes derived from cADSCs represent a promising therapeutic approach for treating inflammatory disorders in companion animals. 14.

Following the same approach here we focused our attention int the treatment of Feline idiopathic cystitis (FIC). FIC is a persistent lower urinary tract condition in cats, resembling PBS/IC in women. This disorder poses a significant challenge in veterinary medicine due to its unknown (idiopathic) cause and the difficulties encountered in its treatment.

Based on this groundwork, the present study aims to characterize and elucidate the molecular composition of fADSC-derived exosomes, subsequently applying them in the treatment of cats with FIC. Ultrasound imaging was utilized to evaluate bladder status pre- and post-treatment with exosomes.

## 2. Materials and Methods

### 2.1 Isolation and culture of fADSCs

The study involved 25 cats of various breeds and ages. The study included 12 male cats (5 intact males, 7 neutered males) and 13 female cats (3 intact female, 10 spayed female) all diagnosed with feline idiopathic cystitis (FIC). Breeds were mainly domestic shorthairs (18), along with Norwegian Forest, Ragdoll, Scottish British Shorthairs. The average age across both groups was 6 years. All cats lived predominantly indoors with limited outdoor access and were fed commercial dry food. They had been vaccinated with core vaccines and were regularly treated for internal and external parasites. None of the cats had received medication or antioxidant supplements within the 30 days leading up to the study. Inclusion criteria for the study group required the diagnosis of FIC. Exclusion criteria included the presence of urolithiasis, urethral plugs, or neoplasia. The clinical signs for FIC cats had lasted no more than 48 hours prior to presentation. The group consisted of healthy cats with no previous urinary tract issues, no current clinical signs of urinary tract disease, and normal clinical and laboratory test results.

Feline adipose-derived Stem Cells (fADSCs) were isolated from fresh biopsy of adipose tissue obtained from 25 healthy cats. Tissue was firstly washed with DPBS (Dulbecco's Phosphatase Buffered Saline of Euroclone, Milan, Italy), added with 2% of Antibiotic/Antimycotic Solution 100X (Anti/Anti) (Sigma-Aldrich, Saint Louis, MO, USA) for 15 minutes. After this, adipose tissue was minced and digested with collagenase type II (Sigma-Aldrich, Saint Louis, MO, USA) in Hanks' balanced salts solution (HBSS) with calcium and magnesium (Euroclone, Milan, Italy) at room temperature (RT) for three hours with shaking. The resulted cells were cultured in DMEM Dulbecco′s Modified Eagle′s Medium high glucose (Sigma-Aldrich, Saint Louis, MO, USA) added with 2% of Anti/Anti and 10% Fetal Bovine Serum (FBS) (Thermo Fisher Scientific, Waltham, Massachusetts, USA) inside incubator at 37°C with 5% of CO2. Cellular growth was monitored using Evos XL Core microscope (Invitrogen, Waltham, Massachusetts, USA).

### 2.2 fADSCS-derived exosomes isolation

fADSCs were cultured in a medium composed by DMEM Dulbecco′s Modified Eagle′s Medium high glucose (Sigma-Aldrich, St. Louis, Missouri, USA) supplemented with 1% Antibiotic Antimycotic Solution 100× (Sigma-Aldrich, St. Louis, Missouri, USA) and 10% exosome-depleted Fetal Bovine Serum (Thermo Fisher Scientific, Waltham, Massachusetts, USA) to avoid the presence of serum lipoproteins. After 72 hours, exosomes isolation was performed using Amicon® Ultra-15 Centrifugal Filter Unit with Ultracel-100 regenerated cellulose membrane (cat. no. UFC910024, Millipore, Massachusetts, USA). Cellular medium was centrifuged at 2000 rcf for 30 minutes at +4°C; isolated content of medium was then washed with PBS at 2000 rcf for 30 minutes at +4 °C; at the end exosomes retained by the filter were collected and stored at -20 °C.

### 2.3 Exosome observation with Transmission Electron Microscopy (TEM)

To prepare exosomes for Transmission Electron Microscopy (TEM) they were fixed in 2% glutaraldehyde solution in phosphate buffer (ratio 1:1). Exosomes were then deposited, rinsed, and stained with reduced osmium (1% osmium tetroxide, OsO4, in 1.5% potassium ferricyanide, K3Fe(CN)6, for 1 h on ice) compounds onto a gridded slide following standard protocols. The slide was visualized with a TEM Zeiss EM 910 instrument (Zeiss, Oberkochen, Germany).

### 2.4 Exosome characterization with Tunable Resistive Pulse Sensing (TRPS)

Diameter and distribution size of feline exosomes isolated from Adipose Stem Cells were analyzed with qNano platform (iZON Science, UK). The analysis was performed using a NP150 nanopores and CPC200 calibration particles at 20-mbar pressure. Results were analyzed with the Izon control suite v3.4 software that allows us to make a comparison between the sample and CPC200 as comparative reference.

### 2.5 fADSCs-derived exosome markers

We analyzed small extracellular vesicle (sEV) markers using the Exo-Check™ exosome antibody array (Systems Biosciences, USA) according to the manufacturer's instructions. The markers tested were ALIX, FLOT1, ANXA5, and TSG101, along with two HRP detection controls, a blank spot, and the GM130 cis-Golgi marker for cellular contamination. Experiments were conducted in triplicate. The antibody used are set up for human used since no antibody specific of feline exosomes are present in the market.

### 2.6 Total exosomes RNA extraction, miRNA sequencing and feline-miRNAs comparison with human-miRNAs homologous

Total RNA extraction from the fADSC-derived exosomes was performed using the Cell Culture Media Exosome Purification and RNA Isolation Mini Kit (cat. no. 60700, Norgen Biotek Corp., Thorold, Ontario, Canada), following the manufacturer's instructions. All RNA samples were then stored at -80°C.

Illumina sequencing was used to realize miRNA profiling, which was carried out by Area Science Park (ASP, Trieste, Italy). MiRNA-Seq libraries were performed using the QIAseq miRNA Library Kit (QIAGEN; Hilden, Germany) and sequenced using Novaseq 6000 (Illumina; San Diego, CA, USA) in the 2 × 150 paired-end mode. Identification of miRNAs in the samples was executed using the QIAseq miRNA-NGS data analysis soft-ware V1 and V2 considering Single Read as the read type and Read 1 Cycles 75 as the read cycles.

MicroRNAviewer software was used to search for homologous matches between Human and Felin Catus miRNAs on the conservation degree reported 15. Together to MicroRNAviewer a specific work performed by Laganà *et al.* 16 was exploit to confirm and implement conservation analysis and specific miRNAs nomenclature for Feline Catus species.

### 2.7 Observation and image acquisition of exosome internalization

To label fADSC-derived exosomes, PKH26 Red Fluorescent Cell Linked Midi Kit for General Cell Membrane Labeling was used (Sigma-Aldrich, Saint Louis, MO, USA). A staining solution of diluent C and PKH26 dye was exploited to incubate exosomes. After that, the incubation was blocked using an equal volume of exosome-depleted Fetal Bovine Serum; 10 mL of PBS were added to the solution, to remove the excess staining, and then was centrifuged at 37400 rcf for 30 minutes with an Ultracentrifuge Optima L-70 (Beckman Coulter Inc., Brea, CA, USA), type 70 Ti rotor. At the end, pellet resulted was resuspended in 200 µL PBS. PKH26-stained exosomes were used to treat 20 000 fADSCs previously seeded on 13 mm glass slides for 24h. After treatment, cells were fixed with PFA 4%, blocked with BSA 1% and incubated with Alexa FluorTM 488 Phalloidin (Thermo Fisher Scientific, Waltham, Massachusetts, USA) and 1% Hoechst fluorescent dye (Sigma-Aldrich, St. Louis, MO, USA) in order to label actin and cell nuclei. At the end, all images were acquired using Zeiss Axiovert 200M Fluorescence Micro-scope (Zeiss, Oberkochen, Germany) equipped with a 63x oil objective.

### 2.8 Bioinformatic and statistical analysis

Every miRNA resulted by QIAseq miRNA-NGS data analysis software was selected on reads number. Only miRNAs having at least 200 reads were considered significant. On the final obtained miRNAs list were performed multiple enrichment using two different software: FunRich 17, and miRNet 18. miRNet provided miRNA target gene data using miRTareBase v8.0 databases as reference. MiRNet functional enrichment analysis was performed on Gene Ontology Biological Process database.

Every experiment was performed in triplicate and consequently data were expressed as the mean ± standard deviation (St. Dev.). All miRNet enrichments were reported with a Prism 8.03 software graphical view (GraphPad Software Inc., Boston, MA, USA).

### 2.9 Specimens

The current investigation adheres to the directive outlined in the Official Gazette agreement dated October 17, 2013, titled "Guidelines regarding the minimal health prerequisites for utilizing stem cells in veterinary practice" (OJ General Series No. 277, 11-26-2013). This directive pertains to Multipotent Stromal Cells (MSC) that have not undergone significant alteration, prepared on an irregular basis, for a substance designated solely for autologous application, within a specific animal owned by individuals or entities, contingent upon their informed permission. Consequently, only informed authorization was required, which was obtained. With owner consent 10 client-owned cats were enrolled based on specific criteria including their signalment, medical history, clinical presentations, and ultrasound diagnostic results suggestive of FIC 19.

### 2.10 Ultrasonography

Ultrasonography is the top pick for diagnosing a wide range of urinary bladder disorders. Positioned close to the surface, the bladder offers easy access for imaging, aided by the urine inside providing excellent clarity for visualizing its walls. This method not only allows for measuring bladder dimensions and wall thickness but also offers a direct view of its outer and inner boundaries. Additionally, it facilitates exploration of nearby structures like the pericystic region and lymph nodes, and simplifies cystocentesis procedures under ultrasound guidance.

Located in the lower abdomen, the urinary bladder is best imaged when the subject is lying on its back or side. In dogs, the bladder neck often retreats into the pelvic area, especially when empty, making it tricky to visualize with ultrasound, particularly if obstructed by the pubic bone. Conversely, feline bladders usually stay within the abdomen, making them easier to scan. Following the standard for other abdominal organs, imaging is done from two different angles, usually sagittal or dorsal and transverse. It's preferable for the bladder to be moderately filled for a thorough evaluation of its inner lining and thickness. In cases of minimal bladder distension, mucosal folding can create a nodular surface, mimicking pathological conditions.

## 3. Results

### 3.1 Feline Catus Exosomes Characterization (TEM, Tunable Resistive Pulse Sensing, markers detection)

**Figure 1:**
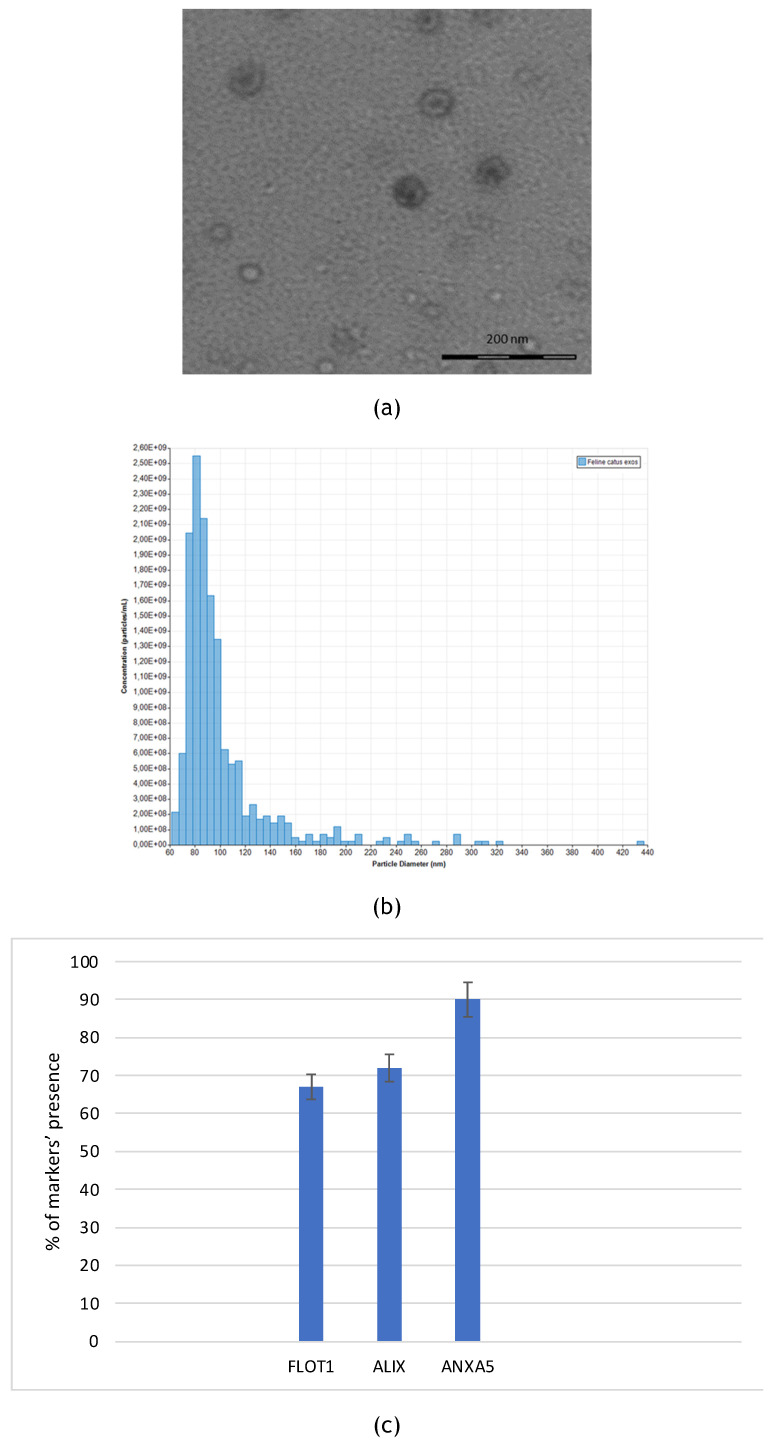
(a) Representative image of fADSC-derived exosomes at TEM. (b) Particle size distribu-tion and concentration of fADSCs-derived exosomes analyzed by tunable resistive pulse sensing: mean diameter of 101 nm (St.Dev.=39,4) mode of 81 nm and average concentration was 1.45e + 09 particles/mL. (c) fADSC-derived exosomes markers: the graphs confirm the presences of typical markers of exosomes as defined by ISEV guidelines, wuch as FLOT, ALIX, ANXA5.

Felin Catus Exosomes were isolated from fADSCs and characterized by multiple laboratory techniques. First, isolated exosomes were observed using transmission electron microscopy (TEM). Resulted extracellular vesicles showed typical bilayer cup-shaped membrane structure. FADSCS-derived exosomes appeared like rounded structures below 100 nm in the transmission electron microscopy (TEM) (Figure 1a). Tunable resistive pulse sensing (RPS) analysis confirmed the dimension of the vesicles: mean diameter of 101 nm and mode of 81 nm and the average concentration was 1.45e + 09 particles/mL (Figure 1b). Employing a semi-quantitative approach, we utilized an exosome antibody array to analyze the protein composition of FADSCS-derived exosomes (Fig. 1C). Notably, we observed positive staining for cell death 6 interacting protein (ALIX), flotillin 1 (FLOT1), annexin A5 (ANXA5). Conversely, no signal was detected for CD63, and CD81, ICAM, EpCAM markers as the array is designed for human samples while our samples are derived from feline sources. There are currently no commercially available reagents specifically designed to identify surface markers of feline exosomes. Consequently, we utilized reagents developed for human markers. Despite the interspecies difference, we observed significant cross-reactivity. As this test is qualitative rather than quantitative, we consider the results to be sufficiently relevant and valuable for our purposes.

### 3.2 Feline Catus Exosomes internalized in fADSCs observation (Confocal Microscopy)

**Figure 2:**
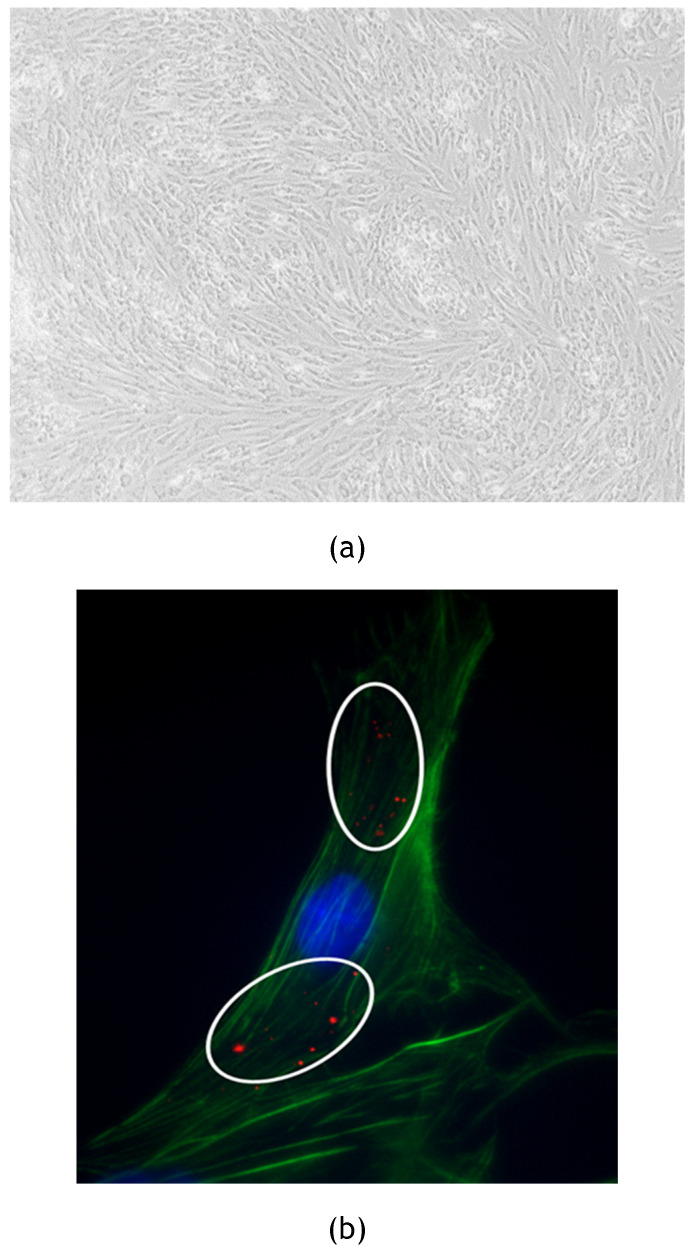
(a) fADSCs observation: fADSCs showed their typical elongated morphology. Picture was captured using 4x objective of EVOS™ XL Core Imaging System. (b) Internalization of fADSCs-derived exosomes by fADSCs. Stained exosomes with PKH26 were internalized by fADSCs and are visible as red spots inside cytoplasmic region of cells (white circles). Zeiss Axiovert 200M Fluorescence Microscope was used to capture the images (63x oil objective). Nuclei of the cells are labeledin blue; Actine filaments are stained in green.

Feline ADSCs were isolated and cultured in order to be treated with exosomes stained with PKH26 membrane dye to observe exosomes internalization. All cells used for the experiment were observed using EVOS™ XL Core Imaging System (Invitrogen, Waltham, Massachusetts, USA) equipped with a 4x objective. FADSCs showed a typical elongated morphology as reported in Figure 2a. After the treatment for 24h, internalized labeled exosomes were observed and captured in pictures using Zeiss Axiovert 200M Fluorescence Microscope (Zeiss, Oberkochen, Germany) as shown in Figure 2b.

### 3.3 Feline and human-miRNAs sequence homologous comparison

**Figure 3:**
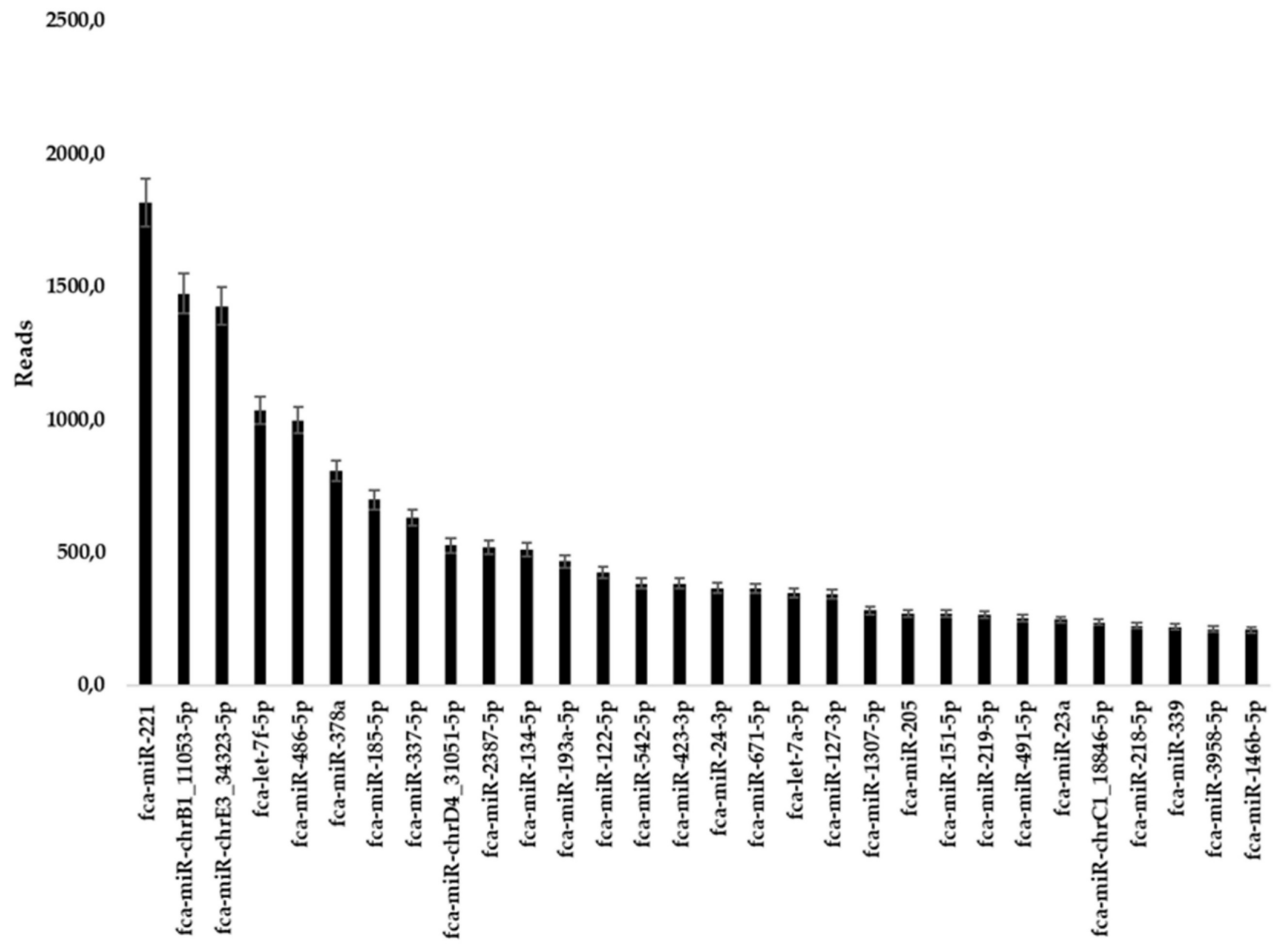
fADSCs-derived exosomes miRNAs content: reads histogram of 30 significant miRNAs resulted from Illumina sequencing.

Illumina sequencing was performed to characterize miRNAs content of fADSCs-derived exosomes. From the analysis 30 miRNAs resulted significant with at least 200 reads as reference value. All miRNAs obtained through Illumina sequencing were reported whit “has” nomenclature due to the alignment with human genome executed by QIAseq miRNA-NGS data analysis software. Using MicroRNAviewer feline miRNAs were found (Table 1 and Figure 3, SI).

### 3.4 fADSCs-derived exosomes enrichment analysis with FunRich and miRNet software

**Figure 4:**
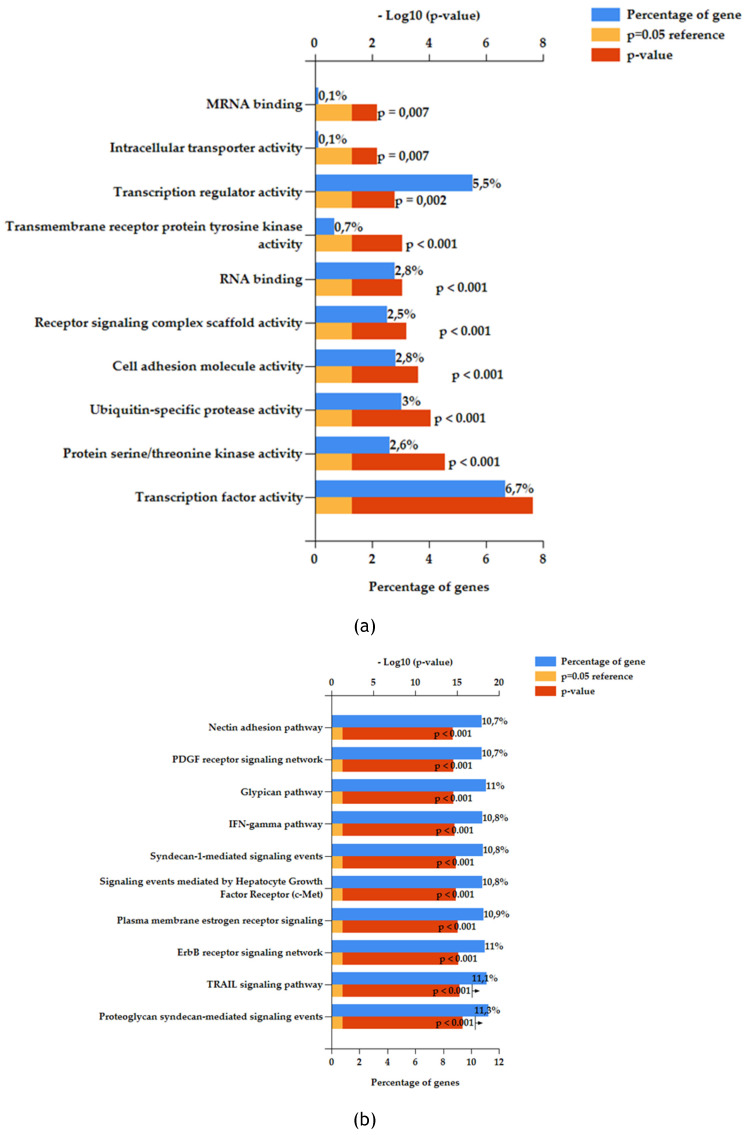
Functional enrichment analysis with FunRich software version 3 on the 30 significant feline miRNAs. (a) Molecular Function enrichment. (b) Biological Pathway enrichment. Percentage of miRNAs involved in each function (blue bar), p-value (red bar) and reference (orange bar);

200 reads were assigned as a cut-off to select significant miRNAs from Illumina sequencing. Under this assumption, 30 miRNAs resulted significant from the analysis as mentioned before. To better understand the possible action of the exosome enriched with these specific miRNAs multiple enrichment analyses were performed. FunRich software was exploited to make Biological Pathway Enrichment (Figure 4a) and Molecular function enrichment (Figure 4b). In both histograms were reported ten most significant pathway enriched. Software uses own reference database.

**Figure 5:**
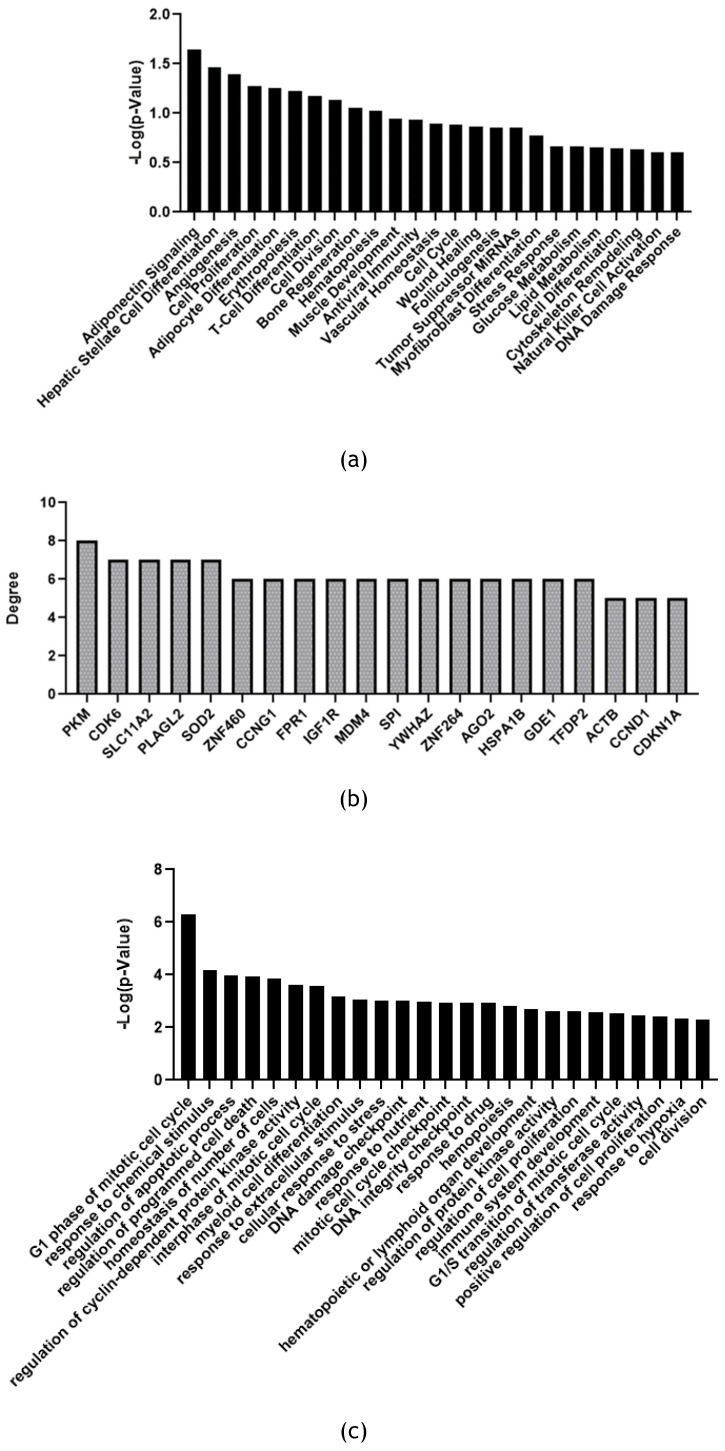
Significant feline miRNAs Functional enrichment analysis with miRNet software. (a) miRNAs function enrichment. (b) 20 main target genes of significant miRNas found with miRNet (c) Biological process enrichment on 20 most significant target genes with Gene Ontology Bio-logical Process (GO:BP) database as reference. Statistical significance is expressed as -Log(p-Value).

MiRNet was used to perform miRNAs function enrichment (Figure 5a) and Biological Process enrichment on the 20 main target genes related to 30 significant miRNAs (Figure 5b and 5c).

### 3.5 Ultrasound

Cystitis, a prevalent pathology affecting the feline urinary bladder, frequently necessitates meticulous evaluation. While overt manifestations may be absent in cases of acute or mild cystitis, chronic presentations often unveil notable alterations in bladder wall integrity, with reductions in echogenicity indicative of edematous changes (Fig. 3 A and C). Notably, wall thickening, a hallmark of cystitis, predominantly localizes to the cranioventral aspect of the bladder but can extend diffusely in severe instances (Fig. 3 A yellow circle and C red circle). It is imperative to exercise meticulous attention to bladder filling status during assessments to mitigate misinterpretation of a structurally intact yet incompletely filled bladder as pathologically thickened. Furthermore, the presence of intraluminal material, such as inflammatory debris, warrants meticulous scrutiny (Fig. 3 A yellow circle and 3 C red circle). Following a therapeutic intervention utilizing fADSCs-derived exosomes, subsequent ultrasound evaluations conducted one month post-treatment revealed a promising restoration of normal bladder architecture in seven feline subjects (Fig. 3 B yellow arrows and D red arrows). Despite this encouraging outcome, definitively attributing the etiology of the condition remained elusive. Consequently, uncertainty persists regarding whether cystitis served as a primary instigating factor or emerged as a sequela to the underlying pathology. These findings underscore the intricate interplay between cystitis and underlying disease processes, underscoring the imperative for continued research to unravel their nuanced dynamics.

## 4. Discussion

It is well known that MSCs are crucial cells involved in tissue regeneration and repair thanks to many peculiarities such as self-renewal capacity, multipotent differentiation, production of growth factor and the immunomodulation function 19.

Exosomes are natural extracellular vesicles (EVs) contain special bioactive molecules for cell-to-cell communications 9. EVs derived from mesenchymal stem cells (MSCs) in detail, attract growing interest for their potential as novel therapeutic agent for many different diseases and they are also interesting as treatment for their lower possibility of immune rejection and good stability and storable opportunity 20-22. Specifically, Adi-pose-derived mesenchymal stem cells (ADSCs) are easy to obtain, and they show all these good MSCs functions 23. So, as reported earlier in the article, ADSCs-derived exosomes maintain similar beneficial effects just like the cells produce them 20. To date, more and more research works are focused on the study regarding exosomes content to improve the understanding about the way of action on the receiver cells.

Translating these notions to veterinary world, exosomes have already become an innovative and potential therapeutic alternative for many disease treatments 24. Present work focused on feline veterinary field with the goal to describe and characterize fADSCs-derived exosomes content in order to better explain their potential beneficial effect.

FADSCs-derived exosomes were isolated and characterized through TEM and TRPS techniques (Figure 1) resulting with a medium diameter of 100 nm. Stained exosomes were also observed after their internalization in fADSCs in order to verify the specific interaction with the cells (Figure 2).

To analyze miRNA content of fADSCs-derived exosomes Illumina sequencing was performed. From this sequencing 30 miRNAs resulted having at least 200 reads to be considered significant (Table 1 and Figure 3). The first work performed on the selected miRNAs list was to investigate the homology between human and feline miRNAs using microRNA-viewer software and previous research 16. To date, no online tools and software are yet available to make functional analysis regarding miRNAome data of Feline Catus species because not equipped with reference database. For this reason, all literature function confirmation and enrichment analysis reported in the present work were realized using corresponding human miRNAs having “hsa” nomenclature in place of “fca”.

Most of the miRNAs discovered inside the feline exosomes resulted associable with positive influence on cellular functions. For example, many miRNAs are reported in literature as tumor suppressor or able to inhibit tumorigenesis like fca-miR-chrE3_34323-5p, fca-miR-486-5p, fca-miR-378a, fca-miR-122-5p, fca-miR-671-5p, fca-miR-3958-5p and fca-miR-1307-5p (human miRNAs corresponding: hsa-miR-3174, hsa-miR-486-5p, hsa-miR-378a-3p, hsa-miR-122-5p, hsa-miR-671-5p, hsa-miR-494-5p and hsa-miR-1307-5p) 25-31. Some other miRNAs instead have been reported to be able to induce proliferative, angiogenic, differentiation and regenerative mechanisms to the cells as fca-miR-221, fca-let-7f-5p, fca-miR-337-5p, fca-miR-542-5p, fca-miR-24-3p, fca-miR-205 and fca-miR-23a (see table 1 to check human corresponding miRNAs) 32-38. Moreover, also miRNAs with anti-inflammatory action like fca-miR-193a-5p and fca-miR-127-3p and miRNAs with positive immune system regulation influence were found (fca-let-7a-5p, fca-miR-chrC1_18846-5p) 39-42. It is also interesting that fca-miR-219-5p (has-miR-6766-3p) has been reported as suppressor of liver fibrosis 43.

As confirmation of the pattern of action related to the significant miRNAs investigated, function enrichments analyses were performed. Through these analyses was demonstrated that the group of miRNAs found inside the exosomes have a potential to induce a lot of positive response from the receiver cells.

From FunRich enrichment (Figure 4a and 4b) it is possible to observe the pattern of molecular and biological function enriched on selected miRNAs as input in the software. Obtained signaling are comparable to a proliferative and development profile. Indeed, we can find many interesting pathways related to development, cell to cell communication, proliferation, and immune system regulation. For example, PDGF receptor signaling network when activated works as stimulator of growth and motility of connective tissue cells 44. While Nectin adhesion pathway is involved in cell-to-cell communication specifically nectin protein interacts with immune receptors 45. Also, immune system molecular functions resulted enriched as IFN-gamma pathway, in line with immunomodulatory action of Mesenchymal Stem Cell. Syndecan-1 is the major syndecan of epithelial cells including vascular endothelium and proteoglycans are major components of the extracellular matrix 46. Both related signaling to these components resulted enriched, highlighting a possible involvement of membrane proteins and extracellular matrix components in response to stimulus induced by the exosomes. All these elements are fundamental in tissue development and regeneration. In addition, Hepatocyte Growth Factor (HGF) signaling via the MET receptor represents an essential signaling for embryonic development and tissue repair. This biological pathway can contribute to tissue repair via stimulating an-ti-inflammatory cytokine production 47.

Based on FunRich functional enrichment analyses, miRNet software was used to confirm and implement data. Using this software, it was possible to make miRNAs function enrichment analysis. Selected significant miRNAs found in fADSCs-derived exosomes resulted involved in many cellular signaling related to differentiation, proliferation, DNA damage response, angiogenesis, tissue development, wound healing mechanisms, and activation/modulation of immune system (Figure 5a). MiRNet is also able to provide target genes of miRNAs list introduced as input in the tool. Choosing to consider the main target genes resulted from the analysis, it was also performed a Biological Process enrichment analysis on these genes using GO:BP as reference database (Figure 5b and 5c). Biological Process enriched on these target genes resulted consistent and superimposable to previous miRNAs function enrichment data (Figure 5a). Indeed, also on target genes molecular signaling enriched are related to cellular functions like proliferation, DNA damage checkpoint, differentiation, development, cell cycle control, cellular stress and hypoxia response, immune system development and regulation.

The findings from the study on Feline Idiopathic Cystitis (FIC) treatment with exosomes and subsequent ultrasonographic analysis reveal promising outcomes. Notably, a significant proportion of treated cats exhibited resolution of symptoms within a relatively short timeframe, with seven out of ten showing improvement after just 15 days of treatment. Ultrasonographic observations before and after treatment demonstrated marked changes in bladder morphology, including the disappearance of intraluminal material and restoration of mucosal integrity, indicative of successful therapeutic intervention. Although histological confirmation of observed abnormalities was lacking, the consistent improvement across cases underscores the potential efficacy of exosome therapy in managing FIC-related urinary tract abnormalities.

## 5. Conclusion

In summation, this investigation underscores the burgeoning potential of exosome therapy in addressing the complexities of Feline Idiopathic Cystitis (FIC). The observed amelioration of symptoms and discernible enhancements in bladder morphology within a condensed therapeutic timeframe herald encouraging prospects for clinical intervention. Nonetheless, the imperative for expanded investigations, incorporating augmented sample cohorts and histological validations, remains palpable to comprehensively delineate the efficacious mechanisms underpinning exosome-based interventions in FIC management. These initial findings, however, resonate within the echelons of burgeoning evidence, emblematic of exosomes' vanguard role in the therapeutic armamentarium against FIC and its urinary tract concomitants in feline cohorts. Consolidating the collective data, the present study underscores the tantalizing prospects of exosomes derived from fADSCs as avant-garde therapies in the feline veterinary landscape. The discerned miRNA payload within feline exosomes evinces an inherent capacity to orchestrate pivotal pathways, including proliferation, angiogenesis, immunomodulation, and anti-inflammatory cascades, thus orchestrating a holistic regenerative milieu. From this synthesis, emerges a compelling narrative, positioning fADSC-derived exosomes as formidable contenders for pioneering therapeutic modalities in feline patients.

## Figures and Tables

**Figure 6:**
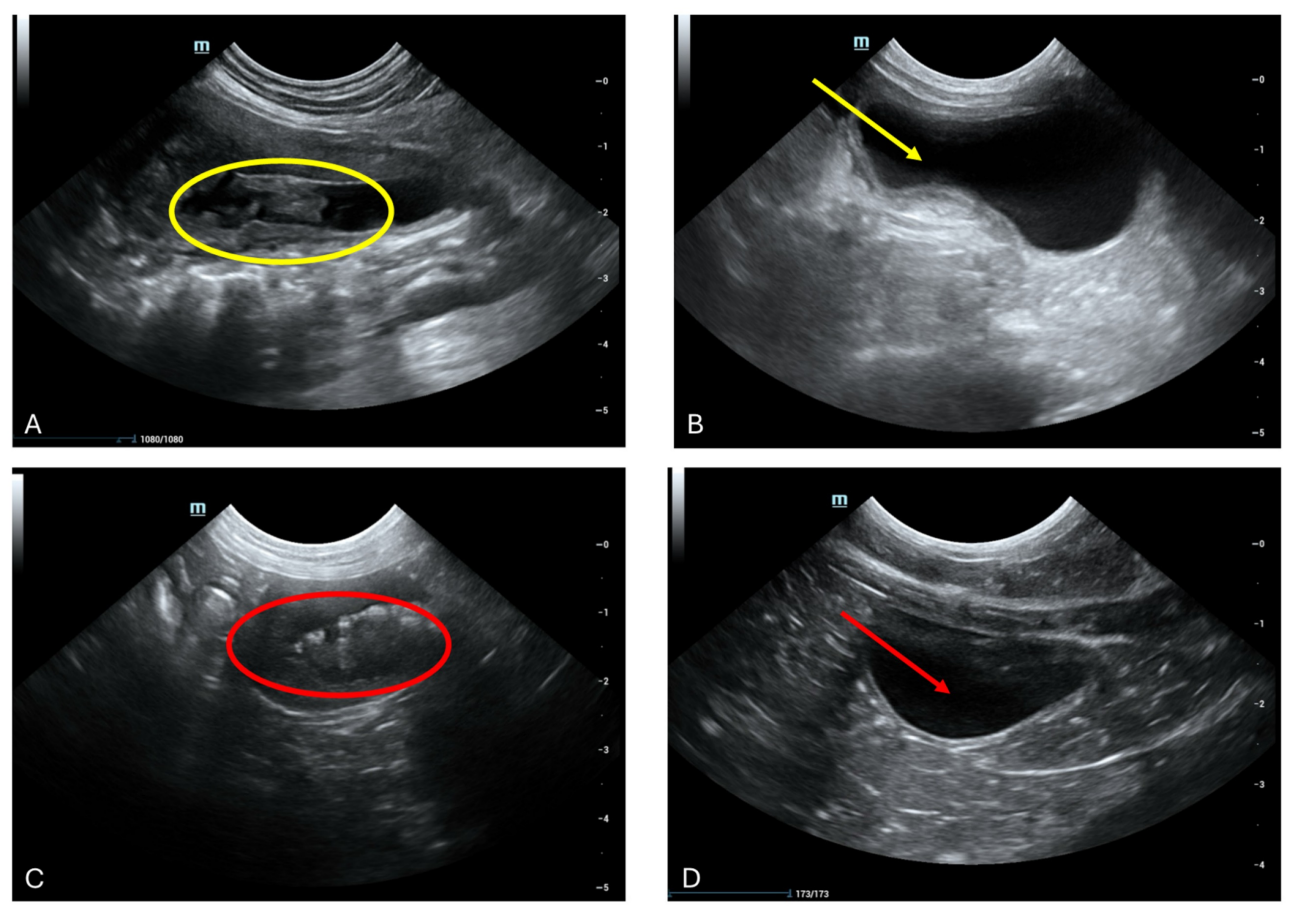
Aberrant intraluminal formations resembling pseudomembranous cystitis, albeit lacking conclusive histological confirmation, were discerned. (a) Ultrasonographic scrutiny of the urinary bladder in a 9-year-old male neutered domestic shorthair cat unveiled marked thickening and irregularities along the cranial bladder wall, concomitant with the presence of intraluminal material (Yellow circle). (b) Following a month of therapeutic intervention, the bladder presented devoid of luminal contents, with a discernibly thin and non-inflamed mucosal lining, indicative of the amelioration of previously identified irregularities (Yellow arrow).(c) Cross-sectional ultrasonographic assessment of the urinary bladder in a 2-year-old female feline unveiled the presence of a dense, highly echogenic sediment within the bladder cavity (red circle), resulting in notable shadowing artifacts during imaging sessions. (d) A 15-day interim follow-up ultrasonographic examination of the aforementioned feline addressing the abnormalities previously detected.

**Table 1:** 30 significant miRNAs resulted from Illumina Sequencing Analysis selected on 200 reads cut-off. Human and corresponding feline miRNAs whit specific reads are reported in the columns.

Human - miRNAs	Feline catus - miRNAs	Mean Reads
hsa-miR-221-3p	fca-miR-221	1815,3
hsa-miR-3130-5p	fca-miR-chrB1_11053-5p	1473,7
hsa-miR-3174	fca-miR-chrE3_34323-5p	1425,3
hsa-let-7b-5p	fca-let-7f-5p	1031,7
hsa-miR-486-5p	fca-miR-486-5p	996,0
hsa-miR-378a-3p	fca-miR-378a	805,7
hsa-miR-4644	fca-miR-185-5p	696,7
hsa-miR-337-5p	fca-miR-337-5p	630,3
hsa-miR-6812-5p	fca-miR-chrD4_31051-5p	524,7
hsa-miR-4533	fca-miR-2387-5p	518,0
hsa-miR-6820-3p	fca-miR-134-5p	509,0
hsa-miR-193a-5p	fca-miR-193a-5p	464,3
hsa-miR-122-5p	fca-miR-122-5p	424,3
hsa-miR-542-5p	fca-miR-542-5p	381,3
hsa-miR-423-3p	fca-miR-423-3p	381,0
hsa-miR-24-3p	fca-miR-24-3p	365,3
hsa-miR-671-5p	fca-miR-671-5p	362,3
hsa-let-7a-5p	fca-let-7a-5p	347,0
hsa-miR-127-3p	fca-miR-127-3p	341,0
hsa-miR-1307-5p	fca-miR-1307-5p	279,7
hsa-miR-205-5p	fca-miR-205	269,3
hsa-miR-611	fca-miR-151-5p	268,0
hsa-miR-6766-3p	fca-miR-219-5p	265,7
hsa-miR-6742-5p	fca-miR-491-5p	250,7
hsa-miR-23a-3p	fca-miR-23a	245,7
hsa-miR-7977	fca-miR-chrC1_18846-5p	236,0
hsa-miR-218-5p	fca-miR-218-5p	222,0
hsa-miR-339-5p	fca-miR-339	219,0
hsa-miR-494-5p	fca-miR-3958-5p	209,0
hsa-miR-7153-5p	fca-miR-146b-5p	206,7
